# Comparison of Novelty Detection Methods for Detection of Various Rotary Machinery Faults

**DOI:** 10.3390/s21103536

**Published:** 2021-05-19

**Authors:** Jakub Górski, Adam Jabłoński, Mateusz Heesch, Michał Dziendzikowski, Ziemowit Dworakowski

**Affiliations:** 1 Department of Robotics and Mechatronics, Faculty of Mechanical Engineering and Robotics, AGH University of Science and Technology, 30-059 Krakow, Poland; jgorski@agh.edu.pl (J.G.); ajab@agh.edu.pl (A.J.); heesch@agh.edu.pl (M.H.); 2 Air Force Institute of Technology, Airworthiness Division, ul. Ks. Boleslawa 6, 01-494 Warsaw, Poland; michal.dziendzikowski@itwl.pl

**Keywords:** novelty detection, data stream, soft computing, gearbox, fault detection

## Abstract

Condition monitoring is an indispensable element related to the operation of rotating machinery. In this article, the monitoring system for the parallel gearbox was proposed. The novelty detection approach is used to develop the condition assessment support system, which requires data collection for a healthy structure. The measured signals were processed to extract quantitative indicators sensitive to the type of damage occurring in this type of structure. The indicator’s values were used for the development of four different novelty detection algorithms. Presented novelty detection models operate on three principles: feature space distance, probability distribution, and input reconstruction. One of the distance-based models is adaptive, adjusting to new data flowing in the form of a stream. The authors test the developed algorithms on experimental and simulation data with a similar distribution, using the training set consisting mainly of samples generated by the simulator. Presented in the article results demonstrate the effectiveness of the trained models on both data sets.

## 1. Introduction

### 1.1. Condition Monioring of Rotating Machinery

Condition monitoring (CM) maintains an essential role in all industrial areas where rotating machinery is applied. Implemented a monitoring strategy allows for cost-efficient maintenance and avoidance of catastrophic failures. In most monitoring systems, assessment of the condition involves vibration signatures’ acquisition. The following actions include extraction of damage-sensitive features and data processing to determine the machine state. More detailed information related to the CM can be provided in references [1,2,3].

There are three major approaches to solve this problem. In the first one, the condition assessment requires only data acquired for an unknown state and a comparison of selected features with proper norms. No information on the previous condition of a particular machine is needed. The described approach is very general and easy to implement. In general, such a system is not sensitive in detecting minor faults that do not cause significant alteration of signal features. However, recent developed real-time algorithms based on eigen perturbation techniques have indicated fault identification of the order 1–5% in real-time. Moreover, studies have also demonstrated the effectiveness of perturbation schemes in a non-stationary time-varying environment with numerical, experimental, and practical applications [4].

The second approach requires a database of signals, including examples acquired on similar machines in healthy and damaged conditions. The obtained data collection allows for designing and training a classifier model for small faults prediction. Unfortunately, such an approach is rarely feasible due to the costly acquisition of signals representing multiple instances of various faults. In work [5], the authors noted a so-called reassembly problem, where an artificial introduction of damage causes structural changes that render pre-acquired baseline signals no longer relevant.

The last approach is a combination of these two. It is based on acquiring signals for one particular machine under monitoring over a long period of its operation. Such a database contains reference patterns for upcoming signals of an unknown state. Any significant alteration from the reference, detected in new data, serves as an indicator of failure. If a single signal feature is used to this end, the approach is called trend analysis. It mostly relies on visual representations, which are sometimes costly to acquire over data transmission systems. For such cases, real-time methods perform analysis online and alleviate data transmission from site to lab, thereby reducing cost. Kalman Filter techniques and eigen perturbation approaches have found extensive applications in this regard [6]. The analysis of multiple features at the same time and the atypical behavior is detected not only in their values but also in relationships between them is referred to as novelty detection, anomaly detection, or outlier analysis [7,8].

### 1.2. Novelty Detection in Condition Monitoring

The article [9] defines novelty detection (ND) as a machine learning class of problems, which is based on identifying new concepts in unlabeled data. The ND idea relies on determining the frontier delimiting the distribution of the initial observations in feature space. If further observations are located within the frontier-delimited subspace, they are considered as coming from the same (normal) population as the initial observations. Otherwise, they are labeled as novelty with given confidence in the assessment.

Various approaches related to the designation of the normal subspace boundaries were developed. Some of the methods are based on the approximation of unknown probabilistic density function (pdf) by known density functions. The most widely used function is Gaussian distribution which was applied inter alia in works [5,10,11,12,13]. Others employ as a boundary the distance threshold from the initial observations. In the articles [12,14,15,16] novelty detection system were implemented based on Euclidean and Mahalanobis distances as metrics. For several presented literature applications, storage of reference observations is required. However, recently developed recursive canonical correlation analysis algorithm do not require a reference for accurate assessment [17]. Another approach to ND also relies on a distance metric, but this time the distance is not measured from reference points stored in memory but from a sample’s reconstructed version [5,18,19,20,21]. In the last quoted approach, the boundary is defined by a trained model. Their location concerning the developed frontiers determines the membership of the data sample under investigation. The most widely used models in this class are Support Vector Machines (SVMs), which were applied by [22] for the online damage detection system.

ND models found applications in condition monitoring for maintanace of rotating machinery. In articles [5,13,18,22,23,24,25] novelty detection algorithms were presented for gearbox monitoring. Other applications include bearings fault detection [14,19,26,27], maintenance support system for gas turbines [10,11,28] and rope inspection [21]. The solutions presented in these articles are, however, usually at the experimental stage. Majority of solutions is yet to be tested under industrial, practical conditions.

### 1.3. Contribution and Organization of This Paper

The task of ND in condition monitoring will have a vital role in the following years and still does not have a reliable solution. Despite the many implementations presented in the previous chapter, the industrial standard for condition monitoring is dominated by determining the structural condition based on analyzing the frequency spectrum content and comparing the values of the calculated indices with the norms [29,30]. This is due to the lack of clearly defined standards for the application of algorithms supporting structure condition assessment. A general framework has been proposed in the literature [31]. The presented solutions guide the system development, but it requires samples for different structure states. Collecting such a set for a specific machine is problematic because it requires operation with damage. Therefore, it becomes reasonable to use ND algorithms, which may not adapt fully to the described framework.

To answer the problem, the article is devoted to verifying and comparing the efficiency of four different ND methods in condition monitoring for a parallel gearbox. Authors implement a data-distribution-based (DDB) method, the nearest neighbor (NN), the online novelty and drift detection algorithm (OLINDDA), and a model-based ensemble of classifiers. The article is a continuation of the analysis presented in [5], where the tests of different ND models were conducted on data from the epicyclic gearbox. That paper focused on detecting faults that were artificially introduced into the analyzed structure without considering the damage severity on the detection threshold.

This paper covers the described problem by introducing different types of damage with variable severity into acquired signals. The algorithms process them sequentially, which mimics the signal processing in condition monitoring and allows the introduction of OLINDDA, developed for ND in data streams [32]. Another novelty is the ensemble built using an Overproduce-and-Choose methodology. The verification is performed based on the simulation data that covers a real object’s model and then on the object itself.

The remainder of the paper is organized as follows: The first section describes methods used for detection of novelty in this work, the next two sections provide an evaluation based on simulation and experimental data, including their description, signal processing, feature extraction, and results discussion. Finally, the last section summarizes and concludes the article.

## 2. Novelty Detection Models

### 2.1. Unidimensional Distribution-Based (Uddb) Approach

**Figure 1:**
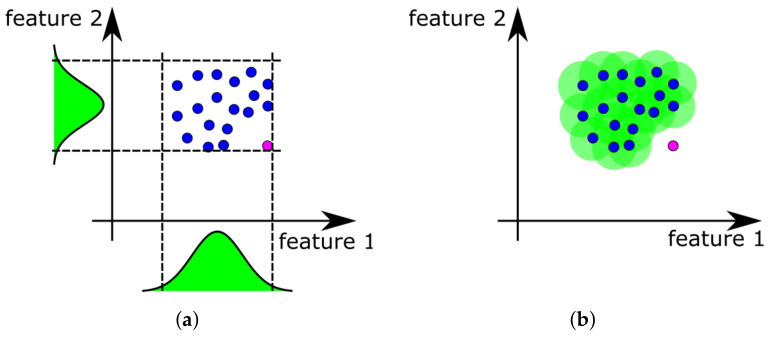
(**a**) The idea of a unidimensional distribution-based model with selected distribution shapes and a threshold for two-feature space and (**b**) the idea of nearest-neighbor model with highlighted normal region.

In this approach, for each data feature, the probability distribution is estimated. The simplest and most commonly applied solution is the assumption of a normal distribution of measured data. The values of the average $\bar{x}$ and standard deviation σ are calculated from training data. From these two quantities, two threshold values for each dimension are set. In general, the threshold values are equals: $\bar{x}+3\sigma$ and $\bar{x}-3\sigma$. Due to this formulation, the model considers many novelty points as normal. The visual concept of the described method is presented in Figure 1a. Blue dots refer to positions of normal data samples in feature space. Dotted lines represent thresholds derived from the assumed type of distribution of these data. Magenta point refers to a novel sample, here detected as normal.

### 2.2. Nearest-Neighbor (Nn) Approach

The Nearest-neighbor (NN) model is based on the unlabeled point’s calculated distances to all points considered during training. If the minimal calculated distance is smaller than the threshold value, then the unlabeled point belongs to the normal class. Otherwise, it is considered a novelty. The threshold value is derived from the calculation of average $\bar{d}$ and standard deviation $\sigma_{d}$ of distances between nearest neighbors in a training set of points. In other words, for each point in a training set, its closest neighbor is located. Distances *d* between these pairs of points are used in threshold calculation. The threshold is set as $\bar{d}+3\sigma_{d}$. The visual concept of the described method is presented in Figure 1b.

The construction of the considered model is very intuitive. However, it requires significant computational effort due to comparison with each database point. Like all distance-based NN methods, it is also sensitive to low data density in feature space and scales poorly to high-dimensional feature spaces.

### 2.3. Online Novelty and Drift Detection Algorithm (Olindda)

**Figure 2:**
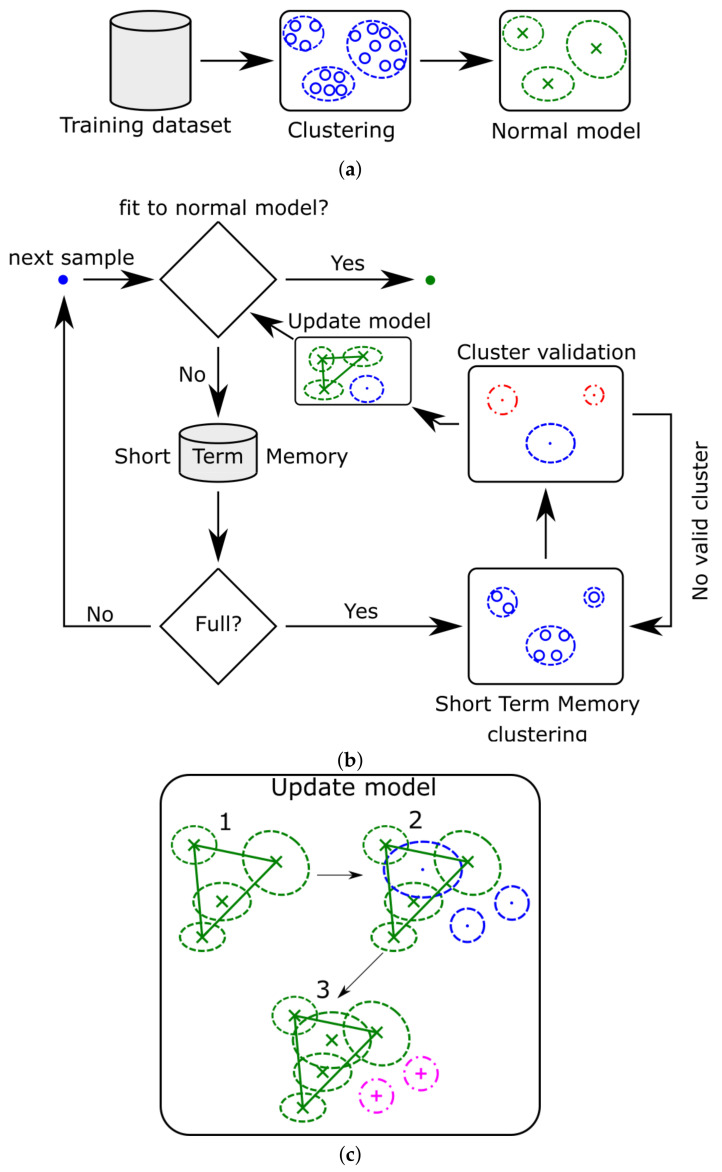
The Online Novelty and Drift Detection Algorithm block diagram for: (**a**) offline stage; (**b**) online stage; (**c**) updated model with convex hull.

The Online Novelty and Drift Detection Algorithm (OLINDDA) is a two-stage algorithm dedicated to detecting new and modifying previously learned concepts from unlabeled samples. The learned concepts are stored in clusters that are represented as hyperspheres with centroid and radius. The idea of OLINDDA is presented in Figure 2.

The training process is divided into two separate stages. In the initial phase called offline presented in Figure 2a, the decision model is derived from an unlabeled data set assuming that all training samples belong to a normal class. During this stage, the training data is divided into clusters by the k-means algorithm. The defined clusters are stored as hyperspheres with cluster centroid $\mu_{i}$ as a center and maximum distance between the centroid and outlier cluster points as a radius. Additionally, for all the defined clusters, the information about minimal cluster density is stored in the form of cohesiveness and representativeness. For this work, the cohesiveness measure was defined as:

(1) $$d_{coh}=\frac{d(x_{j},\mu_{i})}{n(C_{i})}$$

where $n(C_{i})$ is number of samples belonging to cluster $C_{i}$ and $d(x_{j},\mu_{i})$ is the sum of squares of distances between examples belonging to $C_{i}$ and the centroid $\mu_{i}$ defined by:

(2) $$d\left(x_{j},\mu_{i}\right)=\sum_{x_{j}\in C_{i}}{\left(x_{j}-\mu_{i}\right)}^{2}.$$

The representativeness is defined as the number of samples belonging to cluster $n(C_{i})$. More measures have been described in [33].

In the second phase called online presented in Figure 2b, the model receives new unlabeled data samples. If the new example belongs to any of the defined clusters, it is considered as normal. Otherwise is labeled as undefined and stored in short-term memory (STM) of the model. When STM reaches full capacity, the algorithm begins processing undefined points. The analysis employs the clustering of undefined data by k-means algorithm and validation of the received clusters by the criterion of minimal density, derived in the offline phase. There are three possible cases considered for obtained clusters:

- Cluster that fulfills density criteria and is far from normal data is classified as novelty,
- Cluster that fulfills density criteria and is close to normal data is classified as normal-extended,
- Cluster that not fulfill density criteria is classified as noise,

The decision of new clusters membership depends on its centroid’s distance from the centroids of normal clusters. The algorithm applies those points to construct bounding volume. If the centroid of the unlabeled cluster is within the bounding volume, then it is considered normal-extended.

The most common bounding volume is hypersphere, with the center in the centroid of the normal clusters centroids and the radius as the maximum distance between the centroid and normal clusters centroids. However, the hypersphere assumption imposes a constraint of the same distance on every feature dimension. This increases the misclassification of novelty data. Therefore, in this article, the authors applied a convex hull as abounding.

Figure 2c illustrates the procedure for two-dimensional feature space. The convex hull is constructed based on centroid points for normal clusters to cover all those points inside. In the next step, valid clusters (dot-blue) with designated centroid points are introduced to the obtained bounding box. If valid cluster centroid is located in the interior is considered as normal-extended (x green). Otherwise is classified as novelty (+ magenta). The recently added normal-extended clusters will not affect the shape of the designated convex hull.

### 2.4. Auto-Associative Neural Network (Aann)

**Figure 3:**
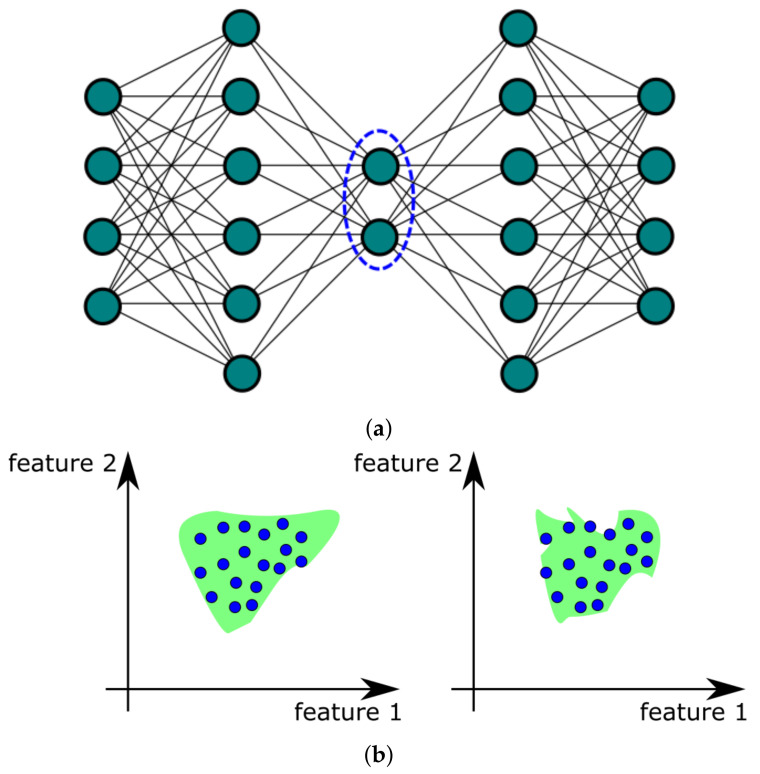
Auto-associative neural network: (**a**) architecture with marked bottleneck; (**b**) different normal regions for trained networks.

The auto-associative neural network (AANN) is a multilayer perceptron (MLP) trained for reproducing input values at its output. The network architecture employs bottleneck to prevent duplicate entries and force generalization: one hidden layer is designed to contain fewer neurons than the input layer. Example of AANN architecture is presented in Figure 3a.

During operation, the unlabeled sample is reconstructed by the AANN model. The error between the sample and its reconstruction is calculated and compared to the threshold value. If the error is smaller than the threshold value, the sample is considered normal. Otherwise, it is labeled as a novelty. The threshold value is described by $\bar{\epsilon }+3\sigma_{\epsilon }$ where $\bar{\epsilon }$ is average and $\sigma_{\epsilon }$ its standard deviation from reconstruction error ϵ for testing dataset.

The training process of AANN is non-deterministic. Therefore, each network, trained on the same training set, gives different normal regions, as presented in Figure 3b. Simultaneous training of several networks and constructing an ensemble, in which their responses are averaged together, producing a more reliable representation of a normal region in feature space [34,35,36].

### 2.5. Ensemble Approach

An ensemble is a set of models whose outputs are combined. The obtained set tends to have a smaller estimation error than the individual models. The ideal ensemble members should be diverse, meaning that their errors should be uncorrelated and accurate, meaning that they provide good-enough classification on their own [37]. The most straightforward approach to ensemble design requires using a group of standard classifiers with diversity enforced by the randomness of their training. Considering this reason, AANNs are natural candidates for ensemble construction due to pseudo-random initializations of initial weights. However, there are no requirements regarding the type of classifiers. Any classifier can be included in the ensemble, provided that it allows for maintaining diverse results.

This basic approach can be further extended using the overproduce-and-choose method [38], in which the actual ensemble is built from a subset of potential ensemble members that were all trained on the subsets of training data. The choice can either be based on the potential ensemble members’ performance on a separate validation dataset [39]. Another selection method based on validation results obtained in the initial training [40]. In this article, the latter was used.

**Figure 4:**
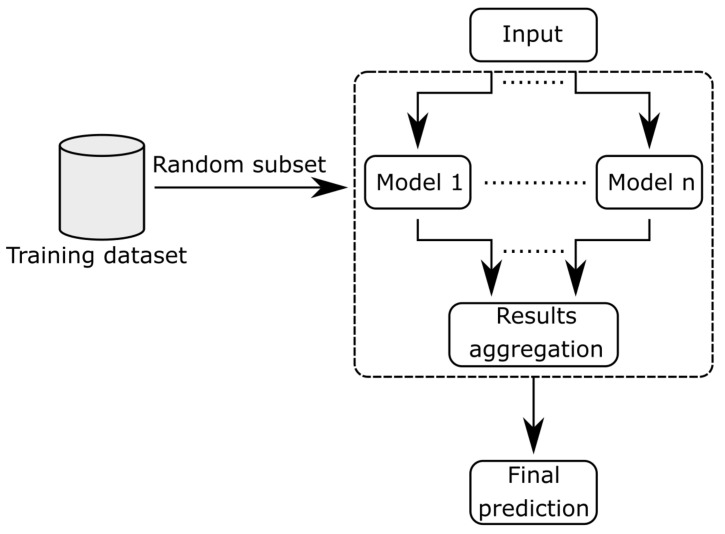
Ensemble block diagram.

The ensemble construction schematic is presented in Figure 4. Models process input features and produce prediction, which is then aggregated. The aggregation is here performed as majority voting.

## 3. Evaluation of Simulation Data

### 3.1. Simulation Data Description

The simulated data are generated from an object representing a drivetrain, which includes a driving shaft associated with referential speed, a one-stage parallel gearbox, a power take-off shaft, and a rolling element bearing (REB). The gearbox is a speed reduction with 23 teeth on the driving shaft gear and 67 teeth on the power take-off shaft gear. The total transmission ratio is 23/67 = 0.34328. The simulated object operates at three nominal speeds of 3000, 4200, and 6000 rpm with a typical minor speed fluctuation of around 12 rpm.

**Figure 5:**
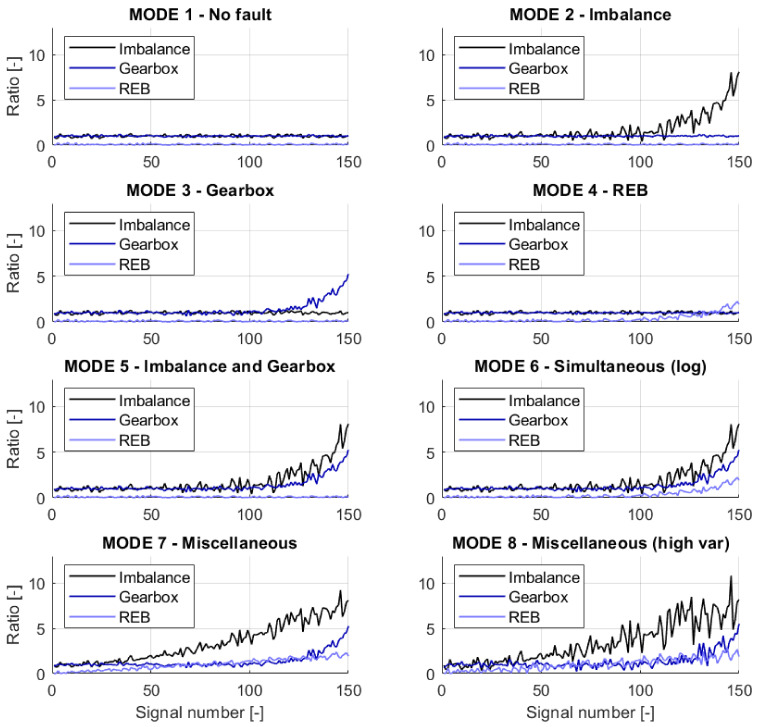
Failure Development Functions (FDFs) for nominal velocity 3000 rpm taken from the book [30].

The simulated data represent eight modes of object structural failures. The list of existing modes is presented in Table 1. Each failure mode is represented by Failure Development Function (FDF) defining the evolution of a particular fault. For the generation of vibrational signals in mode, the simulated object requires 3 FDFs, which indicate the shaft, gearbox, or bearing faults. The FDFs for nominal velocity of 3000 rpm are presented in Figure 5. Each mode contains 150 independent vibrational signals, 10 s. long with a sampling frequency of 25 kHz. Along with the vibrational signal, the object generates a phase marker signal to determine the instantaneous speed.

The simulated model consists of two elements—a synthetic model of vibration signal and a synthetic model of development of particular fault of rotary machinery. Depending on the simulated fault mode, consecutive vibration signals are modified differently. Each vibration signal is constructed as a phenomenological-behavioral model with generalized angular deterministic (GAD) [41] shaft components (AM-FM harmonics) and gearbox components (AM-FM harmonics with multiple double sidebands), as well as generalized angular–temporal deterministic (GATD) [42] rolling-element bearings components (AM-FM cyclo-nonstationary components with additional phase-locked amplitude modulation). Fault development is modeled as a combination of linear, 2nd order polynomial, or exponential growth of amplitudes of individual signal components with relatively low (MODE 2–7) and relatively high variance (MODE 8), as presented in Figure 5. More about the simulated object and the generated signals can be found in the book [30].

### 3.2. Signal Processing and Feature Extraction

During years of research related to condition monitoring (CM), many signal processing and feature extraction algorithms have been proposed. From the fundamental calculation of filtered raw signal root mean square (RMS) to more sophisticated as spectral kurtosis. The detailed description of the various algorithms are described in works [3,30,43].

The choice of signal processing methods for simulation data was based on contextual knowledge. Much of the damage manifests itself in the presence of additional harmonics at specific frequencies. Therefore, spectral analysis was introduced, and the signal was resampled into the order domain based on the phase marker information.

The imbalance is manifested by an increase in the fundamental harmonic amplitude, directly related to the rotational velocity. The raw signal from the generator is supposed to imitate the acceleration waveform. Therefore, to obtain the velocity signal, one must perform the numerical integration operation.

**Figure 6:**
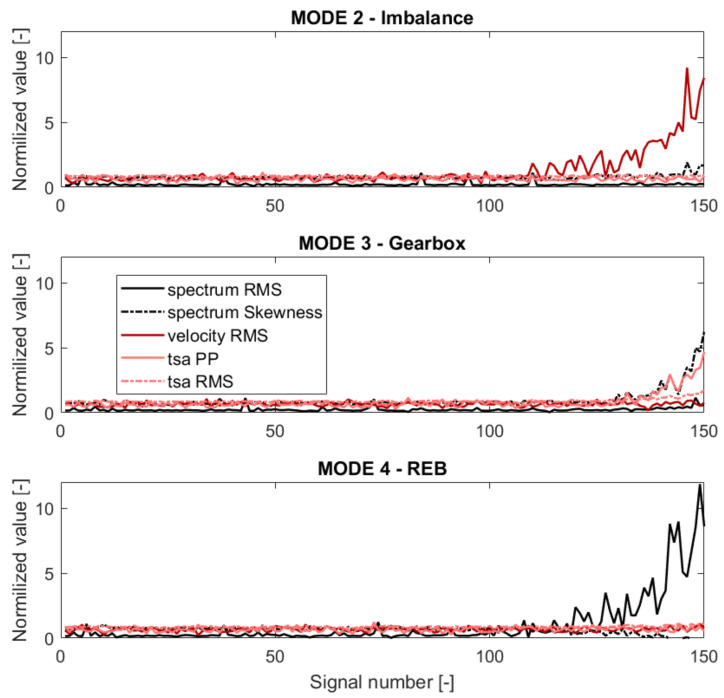
Features trends for the three failure modes described in Section 3.1.

The last applied processing algorithm was time-synchronous analysis, often used to detect gear failure in Vibro-diagnostics. The detailed list of signal processing algorithms and extracted features is gathered in Table 2. The normalized trends for three modes are presented in Figure 6.

### 3.3. Model Training

**Figure 7:**
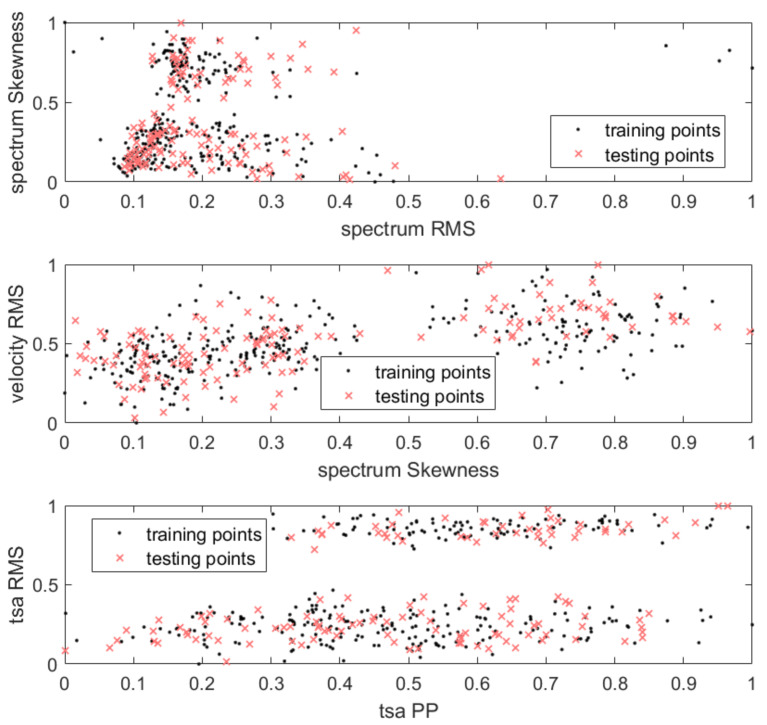
Training dataset points in feature space.

After extracting the features described in the Table 2, authors started developing ND models. For this work, four different models were developed based on algorithms described in Section 2. Those models were:

- Unidimensional distribution-based (UDDB) model,
- Nearest-neighbor (NN) model,
- The Online Novelty and Drift Detection Algorithm (OLINDDA) based model,
- Auto-associative neural networks (AANNs) ensemble,

The ND models were trained on features extracted for the first mode of failure (see Section 3.1), which represented a healthy machine. The training dataset was randomly split in the ratio: 70% for training purposes and 30% for testing false-positive responses. The training and testing subsets in feature space are presented in Figure 7.

The fourth model consisting ensemble composed of 15 AANNs was trained, using the Oveproduce-and-Choose method, in the following manner. For the development of the ensemble, 100 networks were trained on previously split sets. After this process, 15 networks with the best score on the testing set (lowest number of false-positive classification) were selected for ensemble construction.

The models were trained on a computer containing an Intel Core i7-8750H CPU with 16 GB of RAM. The development times for each model are included in Table 3. The training time allocated to Ensemble is distinguishing from other models due to algorithm construction. For UDDB, NN and OLINDDA, exactly one model was developed, while for Ensemble, up to 100 different networks were trained.

### 3.4. Results of Model Evaluation

All trained models were evaluated on simulated vibrational data, which contained failure modes from 2 to 8 (5) for three rotational velocities. Since the results for each rotational speed were similar, the article presents those selected for 3000 rpm. The features in each failure mode were processed sequentially from the 1st to 150th signal as a data stream from a CM system.

**Figure 8:**
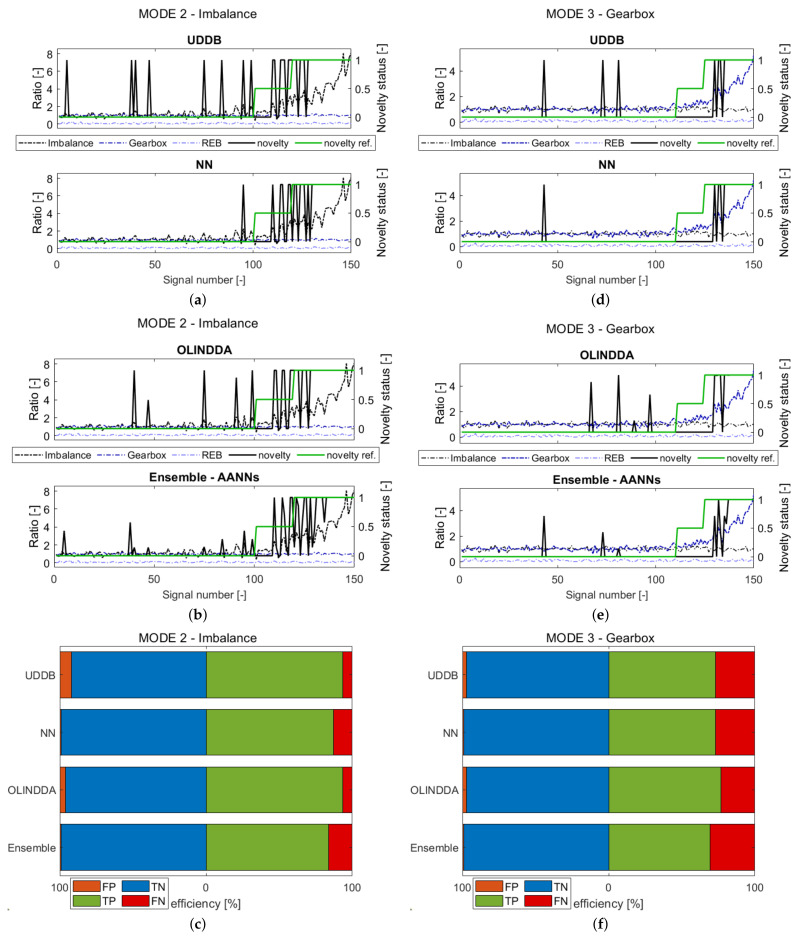
The results obtained for the failure mode 2-Imbalance (**a**–**c**) and mode 3-general gears failure (**d**–**f**). ND waveforms prediction for drive shaft velocity: 3000 rpm (**a**,**b**) or (**d**,**f**); Bar plots containing confusion results for 3000 rpm (**c**) or (**f**).

**Figure 9:**
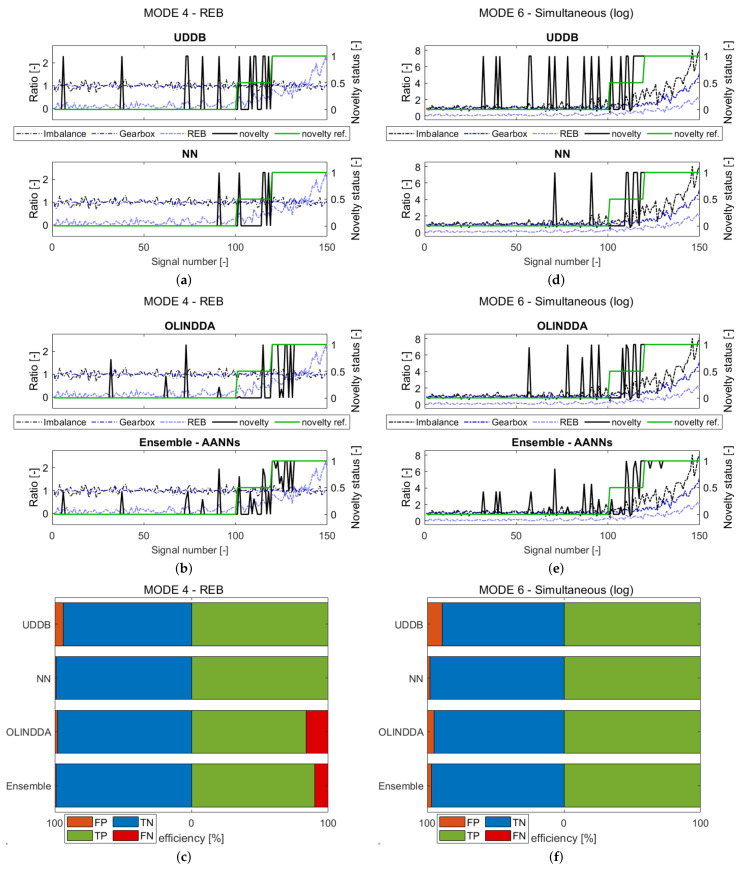
The results obtained for the mode four-rolling element bearing (REB) failure (**a**–**c**) and mode 6-simultaneous failures (**d**–**f**). ND waveforms prediction for drive shaft velocity: 3000 rpm (**a**,**b**) or (**d**,**f**); Bar plots containing confusion results for 3000 rpm (**c**) or (**f**).

In order to present different types of faults, in this subsection only outcome of mode 2, 3, 4 and 6 analysis for one shaft velocity will be discussed (see Section 3.1). The results are presented in Figure 8 and Figure 9.

The four subfigures in each figure (a,b and d,e) reveal sequence of novelty prediction (novelty) and novelty reference (novelty reference) in comparison to the failure development functions (FDFs). In the juxtaposition of the novelty waveforms from these figures, the deviation of models from most samples in the immediate vicinity is present. When such fluctuations occur for an undamaged machine, it indicates false alarms, which generate additional costs.

The rest subfigures in each figure (c,f) contain confusion matrix values in form of bar graphs. All points are divided into 4 groups: True Negatives (TN), False Negatives (FN), True Positives (TP), and False Positives (FP). The values representing the efficiency and false positives percentages for each of the modes are presented in Table 4.

The novelty reference was obtained from FDFs. For FDFs values corresponding to the normal state, the novelty reference is equal 0. For values significantly different from the normal range, the novelty reference is set to 1. For signals in between, the function takes the value 0.5 and, it is not accounted for confusion matrix results. The novelty reference was unknown for the ND algorithms.

The discussion will begin from Mode 2-Imbalance. The results of sequence novelty evaluation are presented in Figure 8a–c. Considering the false positives criterion, the NN and Ensemble would cause the least number of false alarms. Regarding the total efficiency, the best performance in detecting imbalance was obtained by OLINDDA.

Another of the analyzed failures is mode 3-Gearbox, which simulated the generalized gear failure. The waveforms showing the development of damage for 3000 rpm are presented in Figure 8d,e. Considering ND indication fluctuations for the normal state, all presented models reveal similar behavior. Based on Figure 8f, it is impossible to determine which one was the best in terms of the smallest number of false-positive (FP) classifications.

The last type of analyzed failure concerns rolling elements bearings (REB), simulated in mode 4. The waveforms containing the development of damage for 3000 rpm are presented in Figure 9a,b, and the collective results are presented in Figure 9c. Consideration of the number of false alarms exposes a similar prediction rate for all presented models visible in Figure 9a,b. The exception is NN, which manifests in the smallest number of false positives (FPs). The highest ratio of false negatives (FNs) reveals OLINDDA in Figure 9c. Other models also reveal some difficulties with REB failure detection, but comparing to OLINDDA are better. In this statement, the UDDB manifests the highest rate of correct classification.

An explanation of the NN and UBBD improvement is related to the last waveform in Figure 6. The rolling bearing failure occurrence is manifested in the increase of the spectrum RMS indicator. Referring to the algorithm’s feature space, the distance to the samples related to the damage state is distinguishable even for a slowly developing fault. Such behavior allows for proper classification by distance-based algorithms such as NN. The mentioned above damage aspect favors the UDDB algorithm, which assesses states based on one dimension threshold.

The important observation concerning all models is their high sensitivity for detecting this type of damage. Compared to gearbox failure (Figure 8f) or imbalance (Figure 8c), the improvement in performance is significant.

The last results presented in Figure 9e,f show the occurrence of all failures simultaneously. The efficiency of the models achieved in this scenario is the highest one. Here, the model’s efficiency is derived from detecting the failure to which the model is most sensitive. In the case of UDDB and NN, such failure is an inner race fault. The OLINDDA and Ensemble algorithms achieved the highest performance rate when compared to results obtained for each failure separately. The combination of failures activates these algorithms to achieve better performance.

An important aspect regarding the implementation of the algorithms is their computational complexity. Table 5 shows the average model execution time. The shortest execution time is achieved by the UDDB model, while the longest by OLINDDA. The explanation is related to updating the model with new clusters, which takes time but improves the efficiency of structure assessment. Considering both the computation time and efficiency, NN proves one of the highest accuracies with fast execution.

## 4. Evaluation on Experimental Data

### 4.1. Test Bench Description

**Figure 10:**
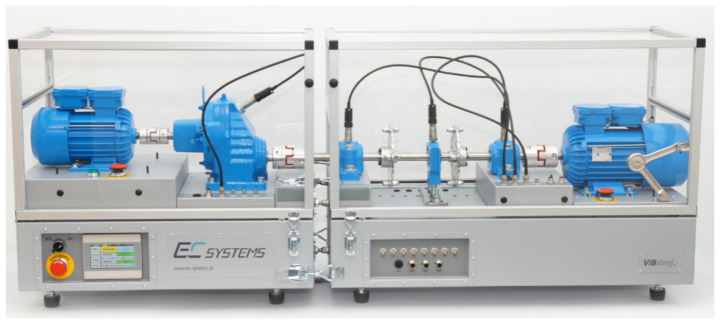
Test bench used in experiments.

Experiments have been conducted on an AMC Vibro VibStand 2 test bench. VibStand 2 test bench configuration is similar to the simulation data generator presented in Section 3. It includes driving an electric motor followed by the parallel gearbox with a reduction ratio of 0.34328. The illustration of the test bench is presented in Figure 10.

Data acquisition was performed with AMC Vibro VibMonitor. As sampling frequency and acquisition time was selected, for each signal, respectively, the following values: 25 kHz and 10 s. Data were gathered with the PCB 333B30 piezoelectric accelerometer mounted on the bearing, similar to the data generator presented in Section 3. Additionally, the phase marker was recorded directly from the AMC Vibro VibStand 2 test bench. A total of 119 measurements belonging to one measurement series were recorded. During signals acquisition, the developing unbalance for the power take-off shaft axis was intentionally introduced.

### 4.2. Signal Processing and Feature Extraction

**Figure 11:**
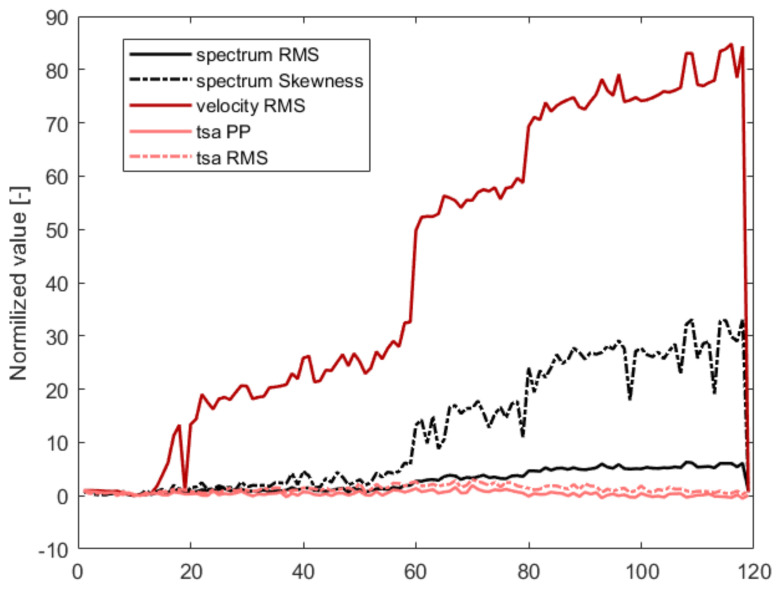
Features’ trends for experimental data described in Section 4.

Due to the similar structure of the data used during the simulation studies, it was decided to utilize the same signal processing algorithms. The reasons behind their application are described in detail in this Section 3.2. The normalized trends calculated for extracted features from measurements are presented in Figure 11.

The presented functions have a similar shape to those obtained for simulation signals in Figure 6. For the normal state of the structure, the values are below 1. When failure emerges, the values of features start to increase. This particular type of damage is characterized mainly by two features: RMS calculated from the velocity spectrum and Skewness derived from the frequency spectrum. This aspect is consistent with the simulation data.

### 4.3. Model Training

**Figure 12:**
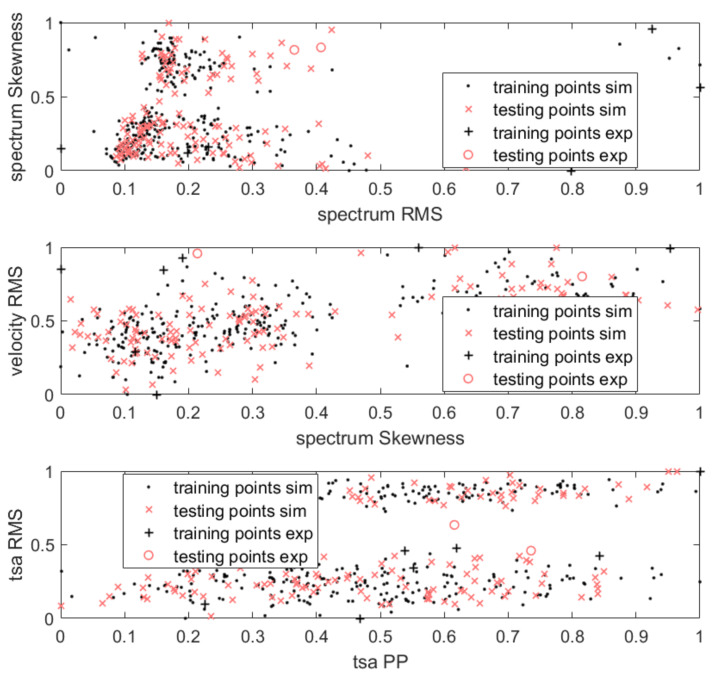
Training dataset points in feature space including points intended for models update.

Because of the low number of normal data samples (only 10 representing the intact state), the training dataset was built by adding to simulation data the experimental samples. The combined samples were divided into a ratio of 70% of training and 30% of testing data. The selected samples concerning previously selected measurement data are presented in Figure 12. The abbreviation sim stands for points from the simulation data, and exp from the experiment.

Models were trained on the same computer. The development times for each model are included in Table 6. The training time allocated to Ensemble is shorter than the initial training time presented in Table 3. It is related to the fact that the procedure retrains only previously selected 15 networks.

### 4.4. Results of the Model Evaluation

**Figure 13:**
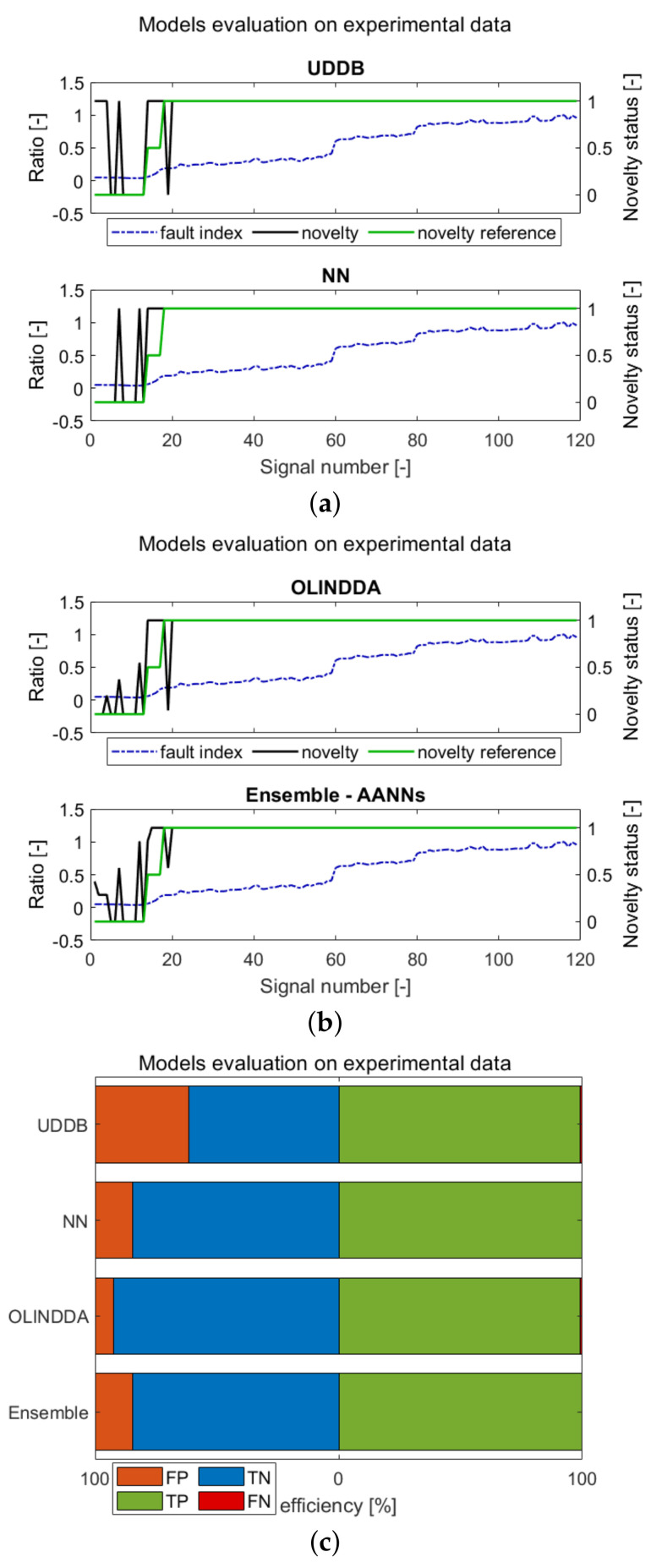
ND results of 6 different models. (**a**,**b**) Waveforms; (**c**) confiusion matrix in form of bar graph.

The retrained models were tested on extracted features from collected data series. The results are presented in Figure 13. The Figure 13a,b contain waveforms presenting model prediction (novelty), reference (novelty reference) and fault index. The Figure 13c represents confusion matrix in the form of bar graphs, representing following numbers: true negatives (TNs), false negatives (FNs), true positives (TPs) and false positives (FPs). The efficiencies and false positives percentages for each of the experimental data have been collected in Table 7.

The novelty reference was obtained by built from fault index before the analyses and was unknown for the ND methods. For fault index values corresponding to the normal state, the novelty reference is equal 0. For values significantly different from the normal range, the novelty reference is set to 1. For signals in between, the function takes the value 0.5 and, it is not accounted for confusion matrix results.

Considering the number of false positives (FP), OLINDDA and Ensemble perform the best. The worst prediction is obtained for the UDDB model. The explanation is related to model architecture, where a few additional points do not affect the distribution parameters. Other methods have no such limitations and therefore produce improved results.

Regarding the overall miss-classification rate, the best performance in detecting inbalance was obtained by the Ensemble method. The combination of responses from different models improved the resultant novelty prediction.

## 5. Summary and Conclusions

In this paper, four different ND methods for CM were implemented. The tests were performed on simulation and experimental vibration signals. The algorithms’ inputs consisted of a vector of features calculated from signals by three different signal processing algorithms. Since the simulation signals had a similar distribution of frequency components to experimental signals, they were introduced to the overall training data.

The data included in the article contained three types of damage: unbalance, gear meshing problem, and bearing failure. All the algorithms scored the lowest in detecting gear damage. This fact is a consequence of the lowest increase in the values of the features. At a low damage severity, sample values did not differ from those obtained for the normal condition.

Worth considering are the results obtained for the shaft unbalance in the drive chain. As described in the paper, the results revealed that all algorithms performed equally well with the simulation and experimental data. It justifies the use of data generators for model preparation. Especially since collecting data from a damaged object is problematic and can cause permanent damage.

The paper also compares the presented models by relating them to the problems encountered in CM. The serious problem is false alarms reported for an undamaged machine. In this category, the best models were NN and Ensemble, which obtained the lowest number of false positives (FPs). Considering the number of effectiveness, NN reached better results. The ensemble model was associated with a relatively high proportion of false-negative (FNs) samples. The calculation costs criterion favors the NN model.

The OLINDDA detector results are ambiguous. The mentioned detector obtained the lowest number of misclassifications on experimental data (low number of false-positive (FPs) and false negatives (FNs). However, regarding the simulation data results, a different view of the OLINDDA detector emerges. Simultaneously, it is the most effective for one type of failure and the worst for another. The algorithm is additionally the most computationally expensive of those discussed in the article.

The worst performance from tested ND models presented a UDDB detector. Considering all the presented results, the mentioned model reported the highest rate of false alarms. Regarding ND capabilities, the model is mediocre in comparison to others. On the other hand, the computational cost for this model is the lowest, which enables implementation in embedded systems with low computing power. The presentation of UBBD in the article is a reference for other multidimensional algorithms which still have not a well-established place in industrial practice.

## Figures and Tables

**Table 1:** Failures modes of generated data.

Mode nr	Mode Name	Mode Description
1	No fault	no fault in the entire drivetrain
2	Imbalance	logarithmic development of the drive shaft imbalance
3	Gearbox	logarithmic development of the general transmission fault
4	REB	logarithmic development of the REB inner race fault
5	Imbalance and Gearbox	simultaneous development of the drive shaft imbalance and the transmission fault
6	Simultaneous	simultaneous development of all the considered faults
7	Miscellaneous	simultaneous development of all the considered faults with different functions
8	Miscellaneous (high var)	simultaneous development of all the considered faults with different functions and high variance

**Table 2:** Signal processing algorithms and extracted features.

Signal Processing Methods Chain	Extracted Features
linear detrending-signal resampling-spectrum	RMS, Skewness
linear detrending-highpass filtration (10 Hz cutoff)-integration	RMS
linear detrending-time synchronous analysis-spectrum	Peak to peak (PP), RMS

**Table 3:** The computational times for model training.

	UDDB	NN	OLINDDA	Ensemble
training time [ms]	0.52	2.24	7.55	30,523.48

**Table 4:** The false positive percentage (FPc) and total efficiencies percentage (TEc) in failure modes.

Failure Mode	Measure	UDDB	NN	OLINDDA	Ensemble
Imbalance	FPc [%]	8	1	4	1
	TEc [%]	93	93	95	91
Gearbox	FPc [%]	3	1	3	1
	TEc [%]	85	86	87	84
REB	FPc [%]	6	1	2	1
	TEc [%]	97	99	91	94
Simultaneous	FPc [%]	11	2	5	3
	TEc [%]	94.5	99	97.5	98.5

**Table 5:** Model execution time.

	UDDB	NN	OLINDDA	Ensemble
mean execution time [μs]	1.1	76.2	791.9	271.6

**Table 6:** The computational times for model retraining.

	UDDB	NN	OLINDDA	Ensemble
training time [ms]	0.49	2.32	7.65	4612.51

**Table 7:** The false positive percentage (FPc) and total efficiencies percentage (TEc) in experimental data.

Measure	UDDB	NN	OLINDDA	Ensemble
FPc [%]	38	15	8	15
TEc [%]	80.5	92.5	95.5	92.5

## Data Availability

Data used in this study are available on-demand from the corresponding author.

## Abbreviations

The following abbreviations are used in this manuscript:

AANN	Auto-Associative Neural Network
AM	Amplitude Modulation
CM	Condition Monitoring
DDB	Data-Distribution-Based
FDF	Failure Development Function
FM	Frequency Modulation
FN	False Negatives
FP	False Positives
GAD	Generalized Angular Deterministic
GATD	Generalized Angular-Temporal Deterministic
MLP	Multilayer Perceptron
ND	Novelty Detection
NN	Nearest Neighbor
OLINDDA	OnLIne Novelty and Drift Detection Algorithm
SVM	Supported Vector Machine
STM	Short Term Memory
TN	True Negatives
TP	True Positives
